# Ultrafiltration membrane for effective removal of chromium ions from potable water

**DOI:** 10.1038/srep41423

**Published:** 2017-01-30

**Authors:** M. R. Muthumareeswaran, Mansour Alhoshan, Gopal Prasad Agarwal

**Affiliations:** 1 King Abdullah Institute for Nanotechnology, King Saud University, P.O. Box 2455, Riyadh, 11451, SAUDI ARABIA; 2 Department of Biochemical Engineering & Biotechnology, Indian Institute of Technology Delhi, Hauz Khas, New Delhi, 110016, INDIA; 3 College of Engineering, Department of Chemical Engineering, King Saud University, P.O. Box 800, Riyadh, 11421, SAUDI ARABIA

## Abstract

The objective of the present work was to investigate the efficacy of indigenously developed polyacrylonitrile (PAN) based ultrafiltration (UF) membrane for chromium ions removal from potable water. The hydrolyzed PAN membranes effectively rejected chromium anions in the feed ranging from 250 ppb to 400 ppm and a rejection of ≥90% was achieved for pH ≥ 7 at low chromate concentration (≤25 ppm) in feed. The rejection mechanism of chromium ions was strongly dependent on Donnan exclusion principle, while size exclusion principle for UF did not play a major role on ions rejection. Feed pH played a vital role in changing porosity of membrane, which influenced the retention behavior of chromate ions. Cross-flow velocity, pressure did not play significant role for ions rejection at low feed concentration. However, at higher feed concentration (≥400 ppm), concentration polarization became important and it reduced the chromate rejection to 32% at low cross flow and high pressure. Donnan steric-partitioning pore and dielectric exclusion model (DSPM-DE) was applied to evaluate the chromate ions transport through PAN UF membrane as a function of flux by using optimized model parameters and the simulated data matched well with experimental results.

Development of effective and economical techniques for removal of chromium from potable water and wastewater has always been a great interest for researchers. Chromium is an essential component, which holds sixth position in earth’s crust in turns of its availability^1^ and it’s one of the fourteen most noxious heavy metals. Generally, in our environment, Cr (VI) and Cr (III) are predominant, whereas chromate ions dominate in oxidizing condition while chromite ions in reducing conditions. Most surface waters and water streams are well aerated, therefore chromium exists as Cr (VI), since ground water is more reducing due to less aeration, thus chromium takes Cr (III) form in this condition^2^. Chromium (VI) ions are known to be highly toxic as compared to other forms of chromium salts, because they are highly soluble and mobile in eco systems^3^. The Environmental Protection Agency (EPA) also reported the higher concentration of chromate ion in aquatic stream at many regions of America, Nepal, Indonesia and India. In addition, the Central Pollution Control Board, India also reported that contamination of chromate ions in water stream especially 250 times higher than WHO permissible limit 50 μg/l^3^,^4^. The long exposure to chromate ions induce skin allergy and is found carcinogenic for living organisms^4^. In aquatic environment, chromate ion has become one of the most dominant components due to its widespread industrial applications^5^. The usual technique for separation of chromium ions are either precipitation and reduction or ion exchange or adsorption processes, and these traditional technologies have the common disadvantages like poor separation efficiency, high-energy requirements, and production of toxic sludge^6^. Recently, many authors investigated the adsorption and other techniques for chromate ions (Cr (VI)) removal. Like Aijuan Xie *et al*.^7^, studied the separation of chromate ions by redox reaction between Cr (VI) and amino/imino groups on poly (mphenylenediamine)/palygorskite (PmPD–PG). Chowdhury *et al*.^8^ examined the Polyaniline nanoparticles grafted silanized silica gel for chromate ions rejection via adsorption-desorption process by using ion exchange mechanism and separation process controlled by pH. Ayse Gul Yavuz *et al*.^9^ used alkyl-substituted polyaniline/chitosan (sPANIs/Ch-HCl) composite as adsorbent material for removal of Cr (VI) and it showed more than 90% rejection. Moreover, compared to traditional or existing methods, membrane based separation processes are emerging as effective technology for water treatment. Membrane separation process, could be classified as microfiltration (MF), ultrafiltration (UF), nanofiltration (NF), reverse osmosis (RO) which depends on applied pressure and pore size of membrane. Number of authors studied the application of NF membrane in the tannery effluents especially for the separation of Cr (III)^6^,^10^. In addition, A. Okhovat *et al*.^11^, studied the application of RO and NF for the separation of chromite ions from tannery effluents at pilot scale and recovered process water. A. Cassano *et al*.^12^, demonstrated the integrated membrane facility for the removal of chromium ions at 98% for NF membranes but as low as 2.1% for ultrafiltration membranes.

To obtain high rejection efficiency of heavy metal ions, the process was further improved by micellar enhanced ultrafiltration (MEUF) and polymer enhanced ultrafiltration (PEUF) membranes. Wang *et al*.^13^, also used the hollow fiber membranes, which gave the effective chromate rejection ≥95.7% at high alkaline condition (pH 12). MEUF process was found to be more attractive method for chromium species removal because of its selectivity and flux. MEUF were based on surfactant processes, in which synthetic surfactant or bio surfactant were used to reject the heavy metal ions^14^. In addition, the separation processes in MEUF were dependent on electrostatic forces, in which chromate ions was bound to the surface of opposite charged micelles. Bohdziewicz^15^ examined the effective removal of chromium ions in PEUF processes, in which chromate ions removed >95% in the presence of hexadecylpyridine chloride complex via 17% of PAN based UF membrane. Recently, Korus and Loska^16^ examined the effective removal of chromium species in PEUF membrane process. In this case, the sodium polyacrylate was bound with chromite and chromate in turn was bound with polyethylenimine, which retained more than 90% of both species. Overall, the removal of heavy metal like chromium through surfactant or ligand complexes or coagulating agent coupled with UF membrane processes resulted in high rejection efficiency and high flux as compared to other conventional membrane processes. However, the disposal of heavy metals coupled with those complex molecules also caused the secondary pollution because of sludge formation^17^.

Sachdeva *et al*.^18^, showed chromate ion rejections ≥90% at basic pH level condition via charged ceramic ultrafiltration membrane. Pugazhenthi *et al*.^19^, examined the modified ultrafiltration charged carbon membrane with a support of macroporous clay, which showed ≥90% rejection of chromate ions at alkaline condition. Polyacrylonitrile (PAN) is one of the most used polymeric membrane material in water treatment because of its chemical stability and hydrophilicity in nature. In literature survey, PAN based membrane (NF and UF) have been used for natural organic matter (esp. humic acid substance), dyes and heavy metals such as arsenic removal application^20^,^21^.

The characterization and modeling of membrane processes were important steps in the understanding and development of new membrane separation processes. Most of the ionic transport models were based on irreversible thermodynamics (IT) or mechanistic approach. Kedem-Katchalsky model and Spiegler-Kedem model^22^ were developed from IT by assuming the flux dependence on to the concentration gradient and pressure gradient. Bowen and Mukthar^23^ described the ionic transport model for nanofiltration, which was known as Donnan steric-partitioning pore model (DSPM). The improved version of DSPM model incorporated dielectric exclusion (DE) principle for the contribution of divalent ions^24^. Recently, A. Szymczyk *et al*.^25^, developed the steric electric and dielectric exclusion (SEDE) model for ionic separation which incorporated the dielectric effect principle (Born solvation energy barrier and image forces contribution) at membrane/solution interfaces.

The present work investigated the rejection of chromate ions from potable water through indigenously developed PAN based ultrafiltration membrane, for the first time^26^,^27^. The surface modified PAN UF membrane morphological property as well as rejection efficiency were compared with nanofiltration membrane. Chromate ions transport through surface modified PAN ultrafiltration membrane was studied by DSPM-DE model to understand the relationship between the parameters of membrane, solutes, and their interaction.

## Results

### Surface and Morphological Properties of PAN membrane

#### Surface Modification of PAN membrane

**Figure 1:**
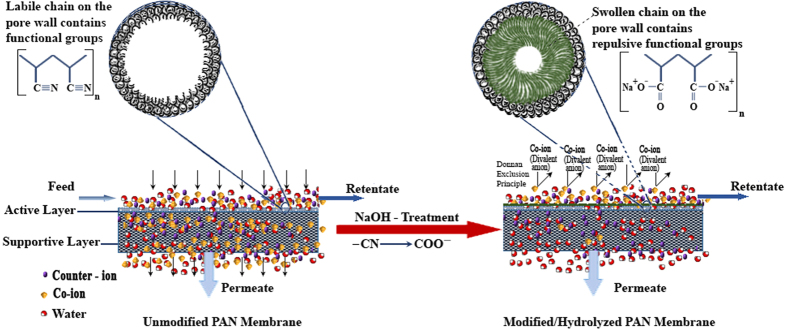
Surface modification and its rejection mechanism of co-ions via PAN UF membrane.

Different coupons of PAN based UF flat sheet membrane were hydrolyzed by 1 N NaOH at 42.5 °C feed temperature using cross-flow velocity of 0.72 ms^−1^ at 1 bar transmembrane pressure. The surface modification and chromate ion rejection mechanism of indigenously developed PAN UF membrane was illustrated in Fig. 1.

From this Fig. 1, one could easily understand the pore size reduction because the formation of COO^−^ on the membrane surface and pore wall. The pure water flux, PEG and model protein rejection studies also confirmed the of pore size reduction. The surface modification of PAN UF membrane was done by standard operating procedure and more details can be found elsewhere^21^,^26^.

#### Model Protein and PEG rejection

**Figure 2:**
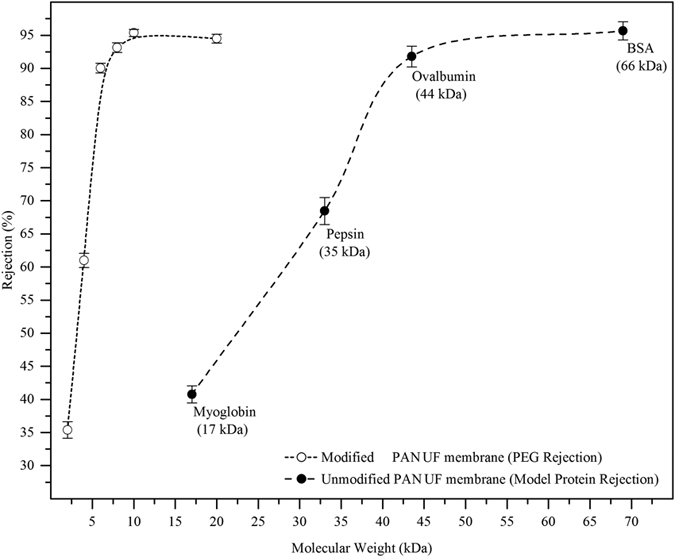
MWCO of unmodified and surface modified PAN UF membrane via Model proteins and PEG Rejection analysis.

The molecular weight cut-off (MWCO) of unmodified and surface modified PAN UF membrane was determined by model protein and polyethylene glycol (PEG) rejection via cross flow mode and the results are shown in Fig. 2. The unmodified PAN membrane had shown the significant passage of myoglobin (17 kDa), pepsin (35 kDa) and more than 90% of rejection was observed in ovalbumin and BSA proteins. In addition, the surface modified PAN membrane showed no transmission of proteins and these membranes were characterized by PEG solution rejection^27^. The unmodified PAN membrane MWCO was found to be ~40 kDa, while modified membrane showed 6–8 kDa. These results inferred that the polymer chain of –COO^−^ groups, on membrane surface, are forcefully moving away from each other due to the repulsive interaction, which led to pore size reduction and morphology change in swollen texture^28^. Moreover, the hydrolysis of –CN functionality of PAN remains in the form of –COO^−^ Na^+^, which may lead to carboxylic group formation. The presence of Na^+^ in the hydrolyzed membrane was similar to unmodified PAN membrane and it was confirmed by Lohokare *et al*.^21^. In addition, the PEG rejections were also used to calculate the mean pore radius (*μ*_p_) and geometric standard deviation (*σ*_p_) of membrane through solute rejection versus solute diameter in log-normal plot^29^; the results were obtained as 1.9 nm, 1.18 nm respectively and more details were provided in Supplementary Information (SI).

#### Effect on membrane roughness

Surface roughness was an important structural parameter because it was being used to study the permeate flux and fouling behavior in the membrane^30^. Table 1 shown that the unmodified and hydrolyzed/modified polyacrylonitrile membrane roughness, permeate flux values were compared with commercially available nanofiltration membrane (NF 200).

These results clearly indicated that after modification PAN UF membrane had less roughness values as well as low volumetric flux as compared to unmodified PAN UF membrane. This was due to the formation of COO^−^ groups in membrane surface which resulted in pore size reduction. In the literature survey, surface roughness value of membrane was also correlated with the permeate flux^31^. It was also reported that the high flux membrane used to obtain high surface roughness values^32^. Moreover, the observed results also indicated that the surface modified PAN UF membranes have higher efficiency in terms of flux (0.25 × 10^−5^ ms^−1^) and surface roughness (3.66 nm) as compared to nanofiltration membrane (NF 200).

#### Effect on pore size distribution and porosity

**Figure 3:**
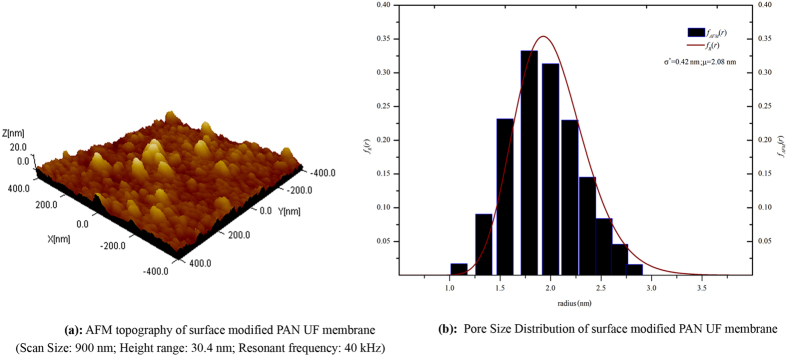
Image topography and pore size distribution of surface modified PAN ultrafiltration membrane.

The membrane pores size and their structures are important factors for ions transport across the membrane or the membrane permeability. Authors also reported that distribution of pores played a vital role on ions separation and permeability of membrane^33^,^34^. The log normal distribution function (Equation 1) was applied to analyze the AFM images for calculating the effective mean pore radius along with pore size distribution, shown in Fig. 3.


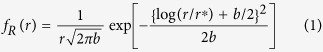


where, 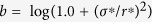 and r^*^- average pore radius of membrane via AFM image.

The effective mean pore radius was found to be 2.01 nm at pH ≥ 7. The variation of water permeability along with pore size distribution (i.e., geometrical standard deviation) of surface modified membrane as a function of solution pH were tabulated in Table 2. As shown in Table 2, pH ≥ 7, the pore size distribution (σ^*^) was found to be less than 0.41 nm with the permeability of ≤0.80 × 10^−11^ ms^−1^ Pa^−1^. Likewise, the permeability (~9.7 × 10^−11^ ms^−1^ Pa^−1^) gradually increased along with pore size distribution (0.98 nm) by decreasing solution pH (up to pH 3). However, after pH 3, the permeability trend got reversed because of iso-electric point (*pI*) of the membrane i.e., the membrane charge converted as negative to positive which was also confirmed by tangential streaming potential measurements and FTIR analysis.

From these results it can be inferred that, the variation of permeability was due to the wide range of pore size distribution and its porosity as a function of pH.

The porosity of surface modified PAN UF membrane assessed by AFM images by using equation (2) and the data’s were shown in Table 2.


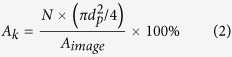


where, *N* –number of pores assessed by AFM image and *d*_*p*_ –mean diameter of pore. At basic condition, the porosity was found to be 0.65 ± 0.04%, but the porosity increased with decrease in pH. This was because of the fact that, at lower pH the conversion of COO^−^ present on the membrane surface to COOH is enhanced. It leads to change in structure of COO^−^ to COOH on the membrane surface as well as pore wall, resulting in increase in porosity which led to the reduction in rejection of ions and increase in permeability of membrane. Moreover, these results, indicated that the porosity is directly proportional to the permeability of membrane. It can be observed in Table 2, that beyond the *pI* point of the membrane, the porosity (1.41%) increased while the permeability got gradually decreased to 4.46 × 10^−11^ ms^−1^ Pa^−1^ at pH 2. This behavior could be due to the change in surface charge i.e., the membrane surface charge converted as negative to positive. This meant that the effective charge density of membrane also played a vital role on ionic separation through modified polyacrylonitrile ultrafiltration membrane.

#### Effect on membrane surface charge

**Figure 4:**
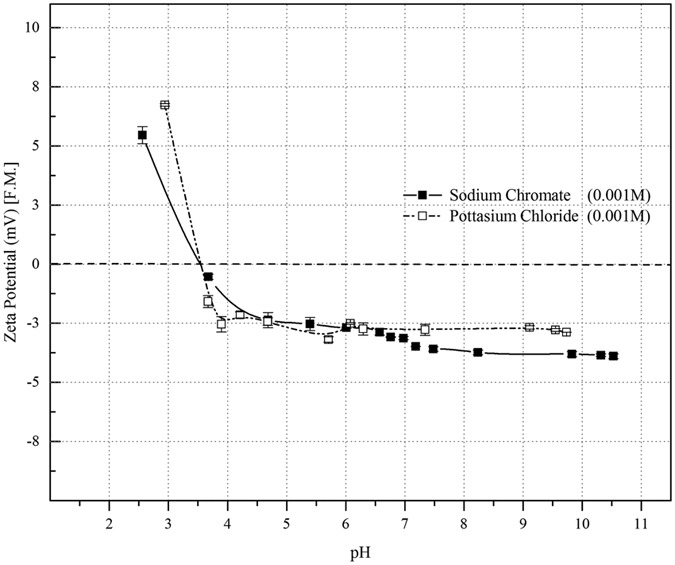
Effect of zeta potential value of hydrolyzed PAN UF membrane as a function of pH for different salts.

The surface charge on membrane surface as well pore wall is an important property for ions transport through the ultrafiltration membrane, because the hydrated radius of the solute (like chromate) is smaller than the pore radius^27^. Different coupons of modified PAN ultrafiltration membrane were analyzed for zeta potential value with the concentration of 0.001 M of KCl & Na_2_CrO_4_ at different pH values and the averaged values were plotted in Fig. 4. The zeta potential values were studied by tangential streaming potential measurements and the detailed experimental procedure could be found elsewhere^34^. As shown in Fig. 4, the marginal effect on zeta potential values were observed on different salts. The isoelectric point (*pI* – 3.6) of membrane was constant with both salts and the variation of zeta potential at alkaline range was −4.0 ± 1.2 mV. Moreover, beyond the isoelectric point (*pI* – 3.6) of the membrane, the zeta values were showed positive which meant the membrane surface charge converted from negative to positive. Experimental observations clearly indicated that the PAN UF membrane zeta values did not appreciably change with respect to salts; however, it would change with feed concentration as a function of given pH^27^.

Gouy Chapman relationship (Equation 3) was used to calculate the electrokinetic charge density (*σ*_*e*_) of the membrane.


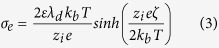


where, *ε, λ*_*d*_, *k*_*b*_, *ζ* are dielectric permittivity, Debye length, Boltzmann constant and zeta potential value respectively. The charge density was assumed to be uniformly distributed in the void volume of cylindrical pores along with the membrane surface^35^. Therefore, the volumetric charge density (*X*_*d*_) was calculated by the following Equation 4,


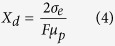


where, *F, μ*_*p*_ – Faradays constant and effective mean pore radius. The PAN UF membrane electrokinetic and volumetric charge density as a function of pH of solute were shown in Table 2. From the observed results, the nano-sized pores had the less electrokinetic charge densities (−4.93 × 10^−4^ Cm^−2^ to 15.6 × 10^−4^ Cm^−2^) but due to less pore size and it led to high volumetric charge densities. Thus, the modified PAN membrane repulse the divalent anionic solutes more strongly as compared to monovalent anionic solutes because of high volumetric charge densities and it was also dependent on concentration of solutes. However, the effective volumetric charge density decreased with the decrease of pH because the isoelectric point of these membranes was located at a lower pH region (Table 2). This phenomenon occurred due to repulsion between ions and the membrane surface.

### Chromate ions rejection

The effect of engineering and chemical operating conditions like concentration of the feed, cross-flow velocity, transmembrane pressure and feed’s pH were studied on surface modified/hydrolyzed polyacrylonitrile ultrafiltration membrane to obtain maximum chromate ions rejection. The rejection efficiency of PAN UF membrane data’s were compared with NF membrane.

#### Effect of feed concentration, Cross flow velocity & Pressure

**Figure 5:**
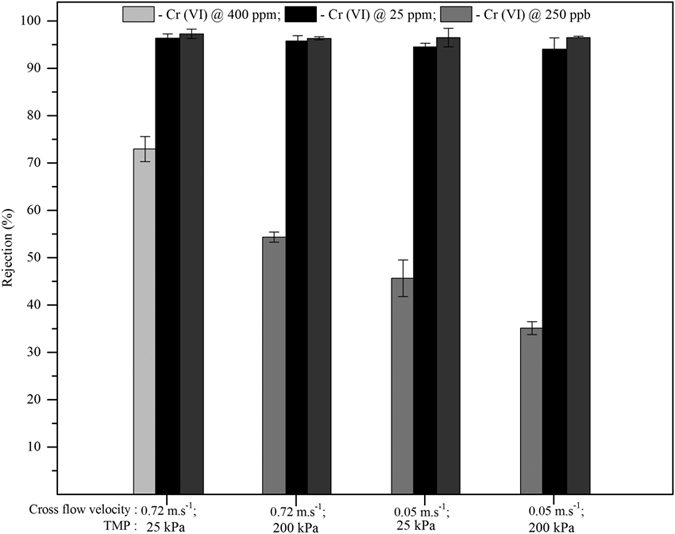
Variation of chromate ion rejection at different feed concentration, cross flow velocity (0.72 ms^−1^ & 0.05 ms^−1^) and transmembrane pressure (25 kPa & 200 kPa) for modified PAN UF membrane [pH: 8.06; temperature: ambient].

To test efficacy of the surface modified PAN ultrafiltration membranes for chromium removal, the experiments were done with different feed concentrations of Na_2_Cr_2_O_4_.4H_2_O (in the range of 400 ppm–250 ppb of chromate ion). The different concentration of chromate ions rejection on the effect of cross-flow velocity and transmembrane pressure were plotted in Fig. 5. As shown in Fig. 5, at pH ≥ 7.0, low feed concentration of chromate (≤25 ppm), the rejection % was found to be ≥95%. The rejection coefficient of chromate ions was reduced to as low as 32% at 400 ppm concentration of chromate ions. This observation could be due to the effect of concentration polarization on membrane surface and it resulted in less rejection % at high feed concentration of chromate ions. For concentration of 25 ppm of chromate (Fig. 5), the cross flow velocity and transmembrane pressure did not change the rejection % of Cr (VI), because concentration polarization (CP) was expected to be negligible at low feed concentration. However, the CP effect was shown at high feed concentration of 400 ppm, the highest rejection was found to be 72% for high cross-flow velocity (0.72 ms^−1^) and low pressure (25 kPa), while low cross-flow velocity and high pressure showed the rejection coefficient of chromate around 32%. This observation matched well with the concept of concentration polarization model (Equation (5)).


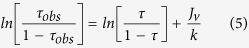


Our previous work^21^ showed that concentration polarization effect on the arsenic rejection at 1000 ppm. However, in the present study for chromate ions rejection, it has been observed that concentration polarization dominated the retention of Cr (VI) ions at a concentration of 400 ppm itself. These observed results clearly indicated that high cross-flow velocity minimizes concentration polarization thus improving the membrane rejection property.

#### Effect of pH

**Figure 6:**
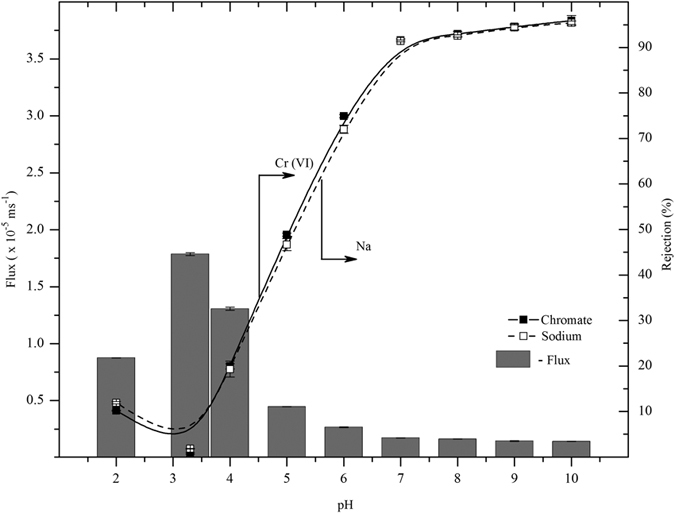
Variation of chromate and sodium ions rejection as a function of feed pH for modified PAN UF membrane [feed concentration: 25 ppm].

**Figure 7:**
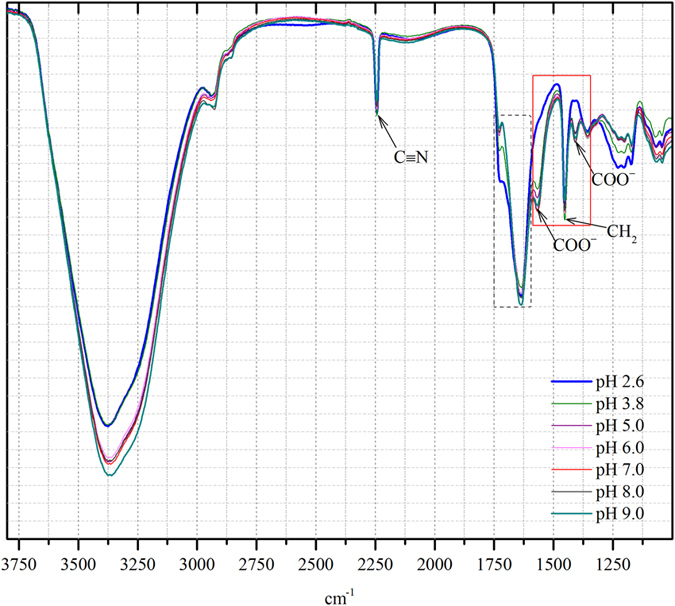
FTIR spectra of surface modified PAN membrane with different feed pH conditions.

The variation in volumetric flux and chromate ion rejection were plotted as a function of pH for negatively charged PAN UF membrane as shown in Fig. 6. For pH ≥ 7, the Cr (VI) rejection was found to be greater than 90% with a flux of 1.62 ± 0.12 × 10^−6^ ms^−1^. Similarly, the sodium ions rejection was greater than 90% for pH ≥ 7, which confirmed the electro-neutrality conditions in the system. In basic condition, chromate ions were repulsed on the negatively charged membrane resulting in a high chromate rejection as per Donnan exclusion principle. For pH < 7, the rejection coefficient decreased to as low as 1% (at pH 3.2), while flux increased to 1.79 × 10^−5^ ms^−1^. This attribute could be explained by the formation of chromate ions species at different pH conditions. The hexavalent chromium may be present in water mainly as chromate 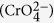, dichromate 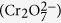, hydrogen chromate 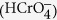, chromic acid 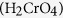 and hydrogen dichromate 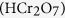 depending on the pH of the solution. The high pH values beyond 7.02, the 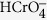 was converted to 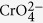
36. Therefore, at high pH (pH ≥ 8) the rejection coefficient of chromate was ≥94% and it was reduced with decreasing pH, because of change in chromium species properties. As shown in Fig. 6, below the isoelectric point (*pI* 3.6), the rejection of chromate and sodium ions gradually increased up to 10% with the decrease of permeate flux to 8.76 × 10^−6^ ms^−1^ at pH 2. This rejection trend was due to positive charge (below *pI*) of membrane i.e. the formation of COOH groups on the PAN, which was confirmed by FTIR analysis (Fig. 7). Moreover, at low pH, the carboxylic groups of PAN UF membrane would be protonated and gave a neutral charge, while higher pH range the carboxyl groups were deprotonated and it produced negative charge on the membrane surfaces.

The functional groups of COO^−^ were found in the peak of 1562 cm^−1^ (strong asymmetrical stretching band) and 1402 cm^−1^ (weak symmetrical stretching band). In addition, the appearance of 1647 and 1728 cm^−1^ peaks are characteristic of the carbonyl (C=O) attached to amine group and hydroxide group^37^. Figure 7, also shown the decreasing of COO^−^ group with decreasing pH (pH 9 - pH 3.8); it also confirmed the disappearance of COO^−^ groups at pH 2.6. From these results it can be inferred that, below the iso electric point of membrane (*pI* – 3.6), the sodium salt of carboxylic group (COO^−^ Na^+^) converted to corresponding carboxylic acid (COOH). Therefore, it can be concluded that the PAN ultrafiltration membranes were efficiently employed in the removal of chromate ions, when feed pH was adjusted to basic condition.

#### Trivalent Chromium ions rejection

**Figure 8:**
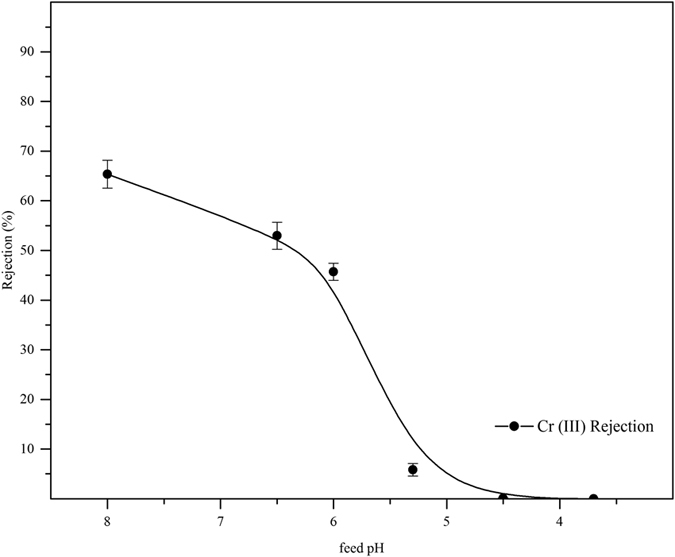
Variation of chromite ions rejection as a function of feed pH for hydrolyzed PAN UF membrane.

Experiments were also performed to test the efficacy of trivalent chromium (Cr-III) ions rejection through surface modified ultrafiltration membrane. Chromium (III) oxide salt at the feed concentration of 100 ppm was used in this study. The rejection of Cr (III) ions as a function of feed pH were plotted as Fig. 8. As shown in Fig. 8, the rejection of trivalent chromium ions decreased (as low as 0.2%) with decreasing pH of feed. This behavior was due to the ionic speciation at a given feed pH. For pH ≥ 5.2, the given ions form was 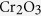 and it reduced to 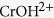 in the pH range of 3.7 to 5. Furthermore, it also moved to Cr^3+^ in highly acidic range (pH < 3.7)^38^. Therefore, the negatively charged membrane repulsive force was very less for Cr (III) ions as per Donnan exclusion principle. Moreover, the nature of the chromium species also influenced the rejection properties of surface modified ultrafiltration membrane. This was because of the fact that the PAN UF membrane, being negatively charged one, allows the monovalent ions to pass through and reject the divalent ions with respect to molecular mass. This explains the typical rejection in the trivalent chromium compared to hexavalent chromium ions in alkaline range.

### Comparative studies between PAN UF and NF membrane

**Figure 9:**
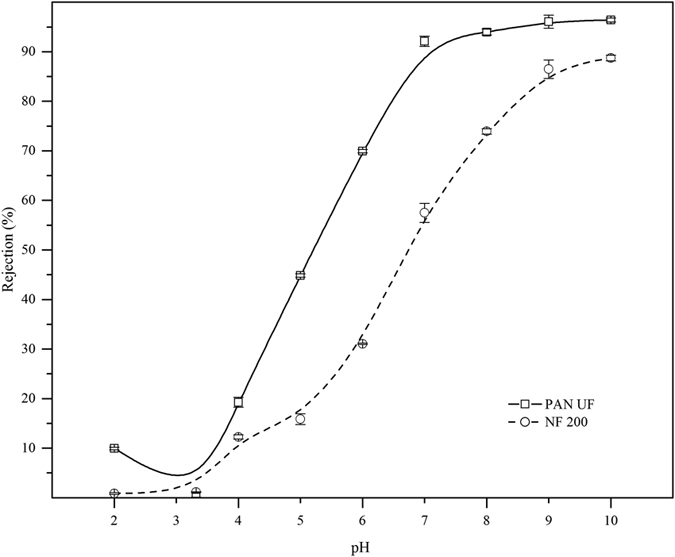
Comparative study of NF200 and modified PAN UF membrane for chromate ion rejection as a function of feed pH [feed concentration: 50 ppm].

The chromate ions rejection efficiency of hydrolyzed PAN membrane were compared with nanofiltration (NF 200) membrane. As shown Fig. 9, the rejection % of chromate ions were more than 95% for hydrolyzed PAN UF membrane at basic condition (pH ≥ 7) with the flux of 1.62 ± 0.12 × 10^−6^ ms^−1^, whereas NF 200 membrane gave only 88% chromate ion rejection with the flux of 1.31 ± 0.2 × 10^−6^ ms^−1^. In addition, the iso-electric point of NF 200 membrane was found to be 2.1, which confirmed that NF membrane (NF 200) has negative charge because the iso-electric point were located in acidic range. The higher rejection % in PAN UF membrane was due to functional groups (COO^−^) present in the pore wall as well as membrane surface, which effectively repulsed the chromate ions. These results also indicated that the surface modified PAN UF membrane showed high performance of chromate ions removal as compared to NF membrane at low pressure. In addition, the results also inferred the functional groups or the structural properties (i.e. intrinsic properties) of membrane were also important for ions transport.

### Modelling of chromium rejection

**Figure 10:**
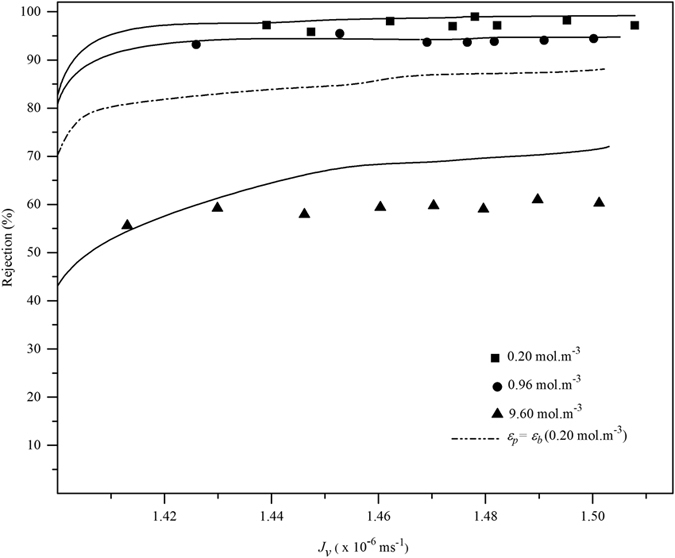
Comparison of experimental and simulated Chromate rejection data (for different feed concentration) as a function of flux (*Jv*); pressure: 200 kPa; temperature: 300 K; *X_d_*: 4 mol m^−3^; *Δx/A_k_*: 1.5 × 10^−5^ m.

Most of ultrafiltration membranes, solute transport works on the principle of size exclusion, however, hydrolysed ultrafiltration membrane, the ion transport was dependent on surface charge on the membrane surface as per Donnan exclusion principle^21^. Moreover, Donnan steric-partitioning pore and dielectric exclusion (DSPM-DE) model has been widely used to study the ionic transport in charged nanofiltration membranes^35^. In this study, the chromate ions transport via surface modified (negatively charged) PAN membrane was evaluated by using one dimensional DSPM-DE model. DSPM-DE model assumes that, the membrane have porous, cylindrical structure and ions transport through the membrane occurred by diffusive mass transport, convective mass transport as well as electro-migration. In addition, the rejection coefficient of chromate ion was calculated by using optimized model parameters as a function of flux. The theoretical description of DSPM-DE model has been given in detail elsewhere^34^,^39^ and brief presentation of the model is given in Supplementary Information. Figure 10, showed the chromate ions rejection of experimental and simulated data for different feed concentration as a function of *J*_*v*_. The optimized model parameters such as charge density (|*X*_*d*_| = 4 mol m^−3^), dielectric constant (*ε*_*p*_ = 48.96), and the membrane thickness to the porosity (*∆x*/*A*_*k*_ = 1.5 × 10^−5^ m) were used to simulate the chromate ion rejection. The results indicated higher rejection (≥94%) of chromate ions with low feed concentration, but they were reduced when the feed concentration increased. A very good agreement was found in between the experimental and simulated results derived from DSPM-DE model. However, at higher feed concentration (9.6 mol m^−3^), the variation of 20% of ionic rejection was found between the experimental and simulated data. This was due to the concentration polarization effect at higher concentration of bulk phase, which was not considered in this study. Calculation were also performed to test validity of standard theory of transport mechanism for Chromate rejection in which dielectric exclusion was not considered (i.e. *ε*_*p*_ = *ε*_*b*_). In Fig. 10, when *ε*_*p*_ = *ε*_*b*_ the rejection coefficient was 80% which did not correlate with the experimental data (at 0.2 mol m^−3^ of feed concentration). These results confirmed that the Donnan steric-partitioning pore model (DSPM) could not explain the rejection data; therefore, the dielectric exclusion (DE) principle was found very important for the modelling of ionic transport through PAN ultrafiltration membrane.

## Discussion

The key objective of this study was to demonstrate a process for chromium rejection from water via indigenously developed surface modified Polyacrylonitrile (PAN) ultrafiltration membrane, as a feasible method of chromium removal from potable water with low operating pressure. Donnan Equilibrium principle played a vital role on chromate ions rejection while size-exclusion principle has little contribution on anions rejection. The solution pH played a significant role on rejection properties of chromate ions and more than 90% rejection was achieved at pH ≥ 7 at a low chromium concentration (i.e. ≤25 ppm) in feed; which approached to beyond detectable limits for concentration of 50 ppb. From the experimental observations, the rejection coefficient was evaluated in terms of flux, charge density, and membrane porosity. Higher rejection rate of chromate ion were obtained with increasing negative charge density and decreasing porosity, flux behavior of the membrane. The concentration polarization effect was found to be negligible at low feed concentration but it became important for feed concentration ≥400 ppm of the solute. The efficiency of PAN UF membrane was tested against the commercial NF membrane; it showed a very high rejection though it could be affected by the concentration polarization at high concentration end. High recovery of chromate ions free potable water was possible with use of polyacrylonitrile ultrafiltration membrane. The predictive model parameters were evaluated in Donnan steric-partitioning pore model and dielectric exclusions (DSPM-DE) to describe the rejection properties of chromate ions through polyacrylonitrile ultrafiltration membrane. The simulated results showed good agreement with experimental rejection data of chromate ions.

## Methods

### Materials used

Different coupons of flat sheet unmodified polyacrylonitrile (PAN) membranes were received from National Chemical Laboratory (NCL) Pune, India^26^. Polyamide nanofiltration (NF 200) membrane was procured from Permionics India Pvt. Ltd. All required chemicals were of AR grade and used without further purification. Ultrapure water with resistivity of 18.2 MΩ was used exclusively in all experiments.

### Experimental Setup

**Figure 11:**
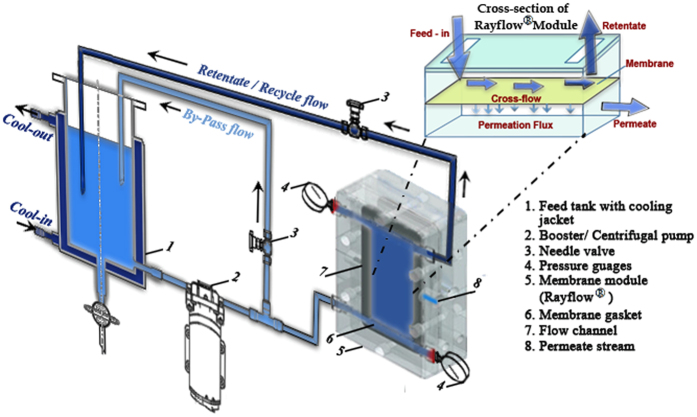
Experimental Setup for Plate and Frame Cross-flow Module.

The plate and frame (RAYFLOW-RAYNOO21) module was procured from TECH- SEP, Groupe Rhone-Poulenc and had the effective membrane surface area of 100 cm^2^ (as shown in Fig. 11). The cross flow velocity of the system was maintained by centrifugal pump, which was also dependent on pump speed, by-pass flow and backpressure. The experiments were performed by total recycle of retentate in a concentration mode in which permeate and retentate was collected separately. The cross flow velocity, transmembrane pressure and feed temperature were maintained at 0.72 ms^−1^, 200 kPa and ambient. The hydraulic permeability of the membranes at given feed concentration was evaluated before and after completion of experiments.

### Anions and Cations Analysis

The chromate concentration in feed, permeate and retentate samples were analyzed by Ion Chromatography (IC 3000) made by Dionex Ltd, USA. Ion Pac As11-HC analytical column (4 × 250 mm) along with Ion Pac AG11-HC guard column (4 × 50 mm) and Ion Pac CS12-HA analytical column (4 × 250 mm), Ion Pac CG12-HA guard column (4 × 50 mm) were used to quantify the anion & cation concentration. 30 mM NaOH and 20 mM methanesulfonic acid (MSA) solutions were used as eluents in this processes for determination of chromate as well as sodium ions. Moreover, for chromate, anionic regenerated suppressor (ASRS) was used and current was set to 160 mA, which was dependent on eluent concentration. For sodium ions, the suppressor (cationic regenerated suppressor) current was set to 70 mA. In addition, for anions, pump flow rate was maintained at 1.5 ml min^−1^, pressure of ≤2500 psi, while for cations 1.0 ml min^−1^, and pressure of 1600 psi. The retention time of chromate and sodium was in 8.2 min, 4.3 min respectively. Trivalent chromium ions were further analyzed by UV-Visible detector, with the mixture of eluents (such as 2 mM PDCA, 2 mM Na_2_HPO_4_, 10 mM NaI, 50 mM CH_3_CO_2_NH_4_ and 2.8 mM LiOH) via Ion Chromatography. In addition, the chromite ions were determined in the form of Cr (III)-PDCA complex with the wave length of 335 nm.

### Atomic force microscopy (AFM)

To determine the membrane structural properties namely porosity, pore size distribution, surface roughness and effective mean pore radius, the different coupons of samples were subjected to chromate ions solution at pH range of 2 to 9. ScanAsyst^®^ mode in atomic force microscopy (Bruker, USA) was used to analyze the samples, (spring constant 0.4 N/m, resonant frequency 40 kHz, 512 × 512 pixel resolution, and image scanning area 900 × 900 nm^2^), which collects force curves at every pixel in the image and it provides faster imaging while retaining high-resolution images^34^. Moreover, surface roughness was dependent on the scanning area, which meant that the roughness value would increase with increasing of scan area of image surface^45^. To obtain the reproducibility, all investigations were performed by same scanning area of samples.

## Additional Information

**How to cite this article**: Muthumareeswaran, M. R. *et al*. Ultrafiltration membrane for effective removal of chromium ions from potable water. *Sci. Rep.* **7**, 41423; doi: 10.1038/srep41423 (2017).

**Publisher's note:** Springer Nature remains neutral with regard to jurisdictional claims in published maps and institutional affiliations.

## Supplementary Material

Supplementary Information

## Figures and Tables

**Table 1:** Surface roughness value and volumetric flux of unmodified, surface modified PAN UF and NF membranes.

S.No.	Membrane	Surface roughness (nm)	Volumetric flux (×10^−5^ ms^−1^)
1	Unmodified PAN_23_UF	9.14	1.28 ± 0.2
2	Modified PAN_23_UF	3.66	0.25 ± 0.06
4	NF 200 (M/s. Permionics)	0.27	0.13 ± 0.2

**Table 2:** Variation of membrane structural properties and charge density of surface modified PAN UF membranes.

pH	2	3.3	4	5	6	7	9
Pore size distribution (σ^*^) (nm)	1.39	0.98	0.74	0.52	0.49	0.41	0.39
Mean Porosity (*A*_*k*_) (%)	1.41	1.15	1.13	0.83	0.72	0.69	0.65
Permeability (*L*_*p*_) ( × 10^−11^ ms^−1^ Pa^−1^)	4.46	9.75	6.60	2.27	1.37	0.83	0.87
electrokinetic charge density (*σ*_*e*_) (×10^−4^ Cm^−2^)	15.6	−0.19	−1.44	−2.52	−3.79	−4.93	—
Volumetric Charge density (|*X*_*d*_*|*) (mol m^−3^)	31.8	0.60	3.60	5.36	8.01	10.44	—
