# Supplementary material for: Ultrafiltration membrane for effective removal of chromium ions from potable water
Source: Sci Rep. 2017 Jan 30;7:41423. doi: 10.1038/srep41423 (PMC5278407; doi:10.1038/srep41423)
Supplement: Supplementary Information [file srep41423-s1.pdf]

Supplementary Information (SI)

Ultrafiltration membrane for effective removal of chromium ions from potable water

M.R. Muthumareeswaran[1,3*], Mansour Alhoshan[1,2], Gopal Prasad Agarwal[3,+],

[1]King Abdullah Institute for Nanotechnology, King Saud University, P.O. Box 2455, Riyadh 11451, (SAUDI ARABIA)
Email: mramamoorthy@ksu.edu.sa

[2] College of Engineering, Department of Chemical Engineering, King Saud University, P.O. Box 800, Riyadh 11421, (SAUDI ARABIA)
Email: mhoshan@ksu.du.sa

[3] Department of Biochemical Engineering & Biotechnology, Indian Institute of Technology Delhi, Hauz Khas, New Delhi- 110016 (INDIA)
Email: gopal@dbeb.iitd.ac.in

1.0 Materials used.

Sodium chromate ($Na_2CrO_4.4H_2O$), Chromium (III) oxide (Cr_2O_3) methanesulfonic acid (CH_4O_3S), Pyridine-2,6-dicarboxylic acid (PDCA), sodium iodide (NaI), ammonium acetate ($CH_3CO_2NH_4$) and lithium hydroxide (LiOH) were procured from Sigma-Aldrich chemicals (USA). Sodium hydroxide (NaOH @ 1 N) solution and sodium phosphate (Na_2HPO_4) were received from Merck (Germany).

2.0 Sample Analysis.

2.1. Model Protein and PEG analysis.

The unmodified and surface modified or hydrolyzed PAN UF membrane molecular weight cut-off (MWCO) was determined by model protein and PEG rejection. The protein sample of permeate and retentate was analyzed by UV-VIS spectroscopy (Cary 60, M/s. Agilent Technologies, USA) at 280 nm and 260 nm respectively. PEG samples were analyzed by total organic carbon analyzer (Sievers 5310C TOC analyzer, M/s. GE Instruments, USA). The rejection coefficient (R_e) of given solute was calculated by using the following equation (1):

$$R_e\ (\%) = \left(1 - \frac{C_p}{C_r}\right) \times 100 \qquad (1)$$

The PEG solute radius were calculated by using the following equation (Eq.1):

$$r = 16.73 \times 10^{-12} (M)^{0.557} \qquad (2)$$

$$R(r) = A_o + A_1 (\ln d_s) \qquad (3)$$

In equation (2), A_0 and A_1 are slope and intercept in log-normal probability plot. Moreover, the mean pore size (μ_p) can be calculated as solute rejection (R) at 50% and pore size distribution at R=84.13%[29].

The mean pore size of membrane and its distribution was calculated by plotting PEG rejection versus solute diameter in log-normal plot as shown in Fig. 1.

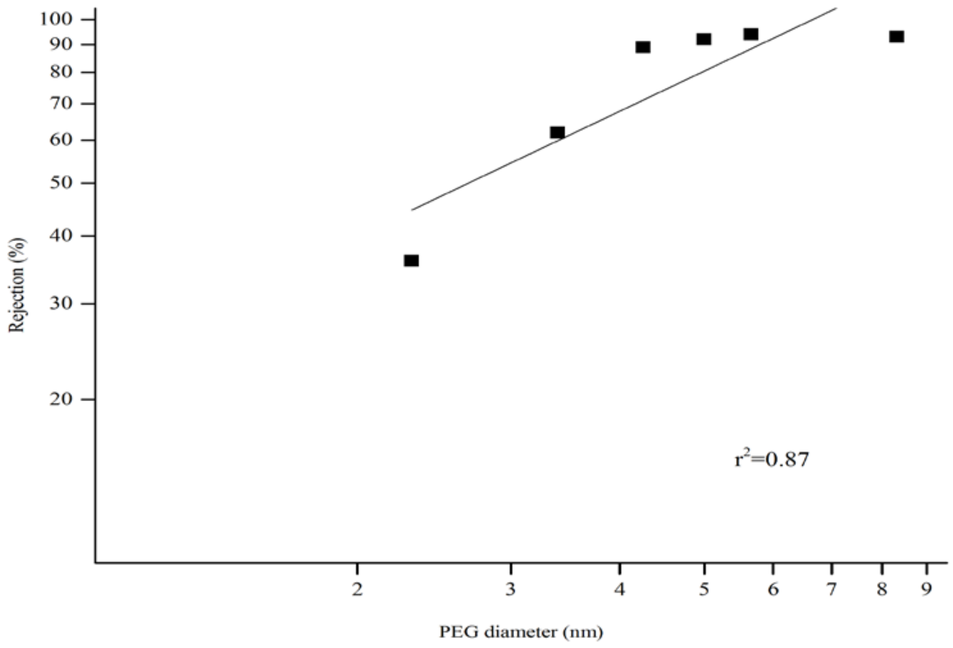

Figure 1: PEG rejection vs PEG diameter for modified PAN UF membrane

2.2. Atomic force microscopy (AFM).

AFM Image analysis was carried out by means of SPIP v.6.0 software and it explored the full details of surface topography like average roughness, root mean square roughness as well as ratio of peak to valley. The average roughness (S_a) parameters from software was calculated by equation (4),

$$S_a = \frac{1}{N}\sum_{j=1}^{N}\left|Z_j\right| \tag{4}$$

where, Z_j is the current Z value, and N is the number of points within the box cursor. Before analyzing the desired images on SPIP software, it was corrected by linewise levelling to reduce the noise. The desired surface topographic images were used to calculate the number of pores by

using SPIP™ V.6.0 image metrology software[40]. Median rank [50%] was applied to arrange the number of pores by ascending order (equation (5)) as it follows:

$$\chi = \left(\frac{k_s - 0.3}{m + 0.4}\right) \times 100 \qquad (5)$$

The trapezium rule (very narrow step size of 0.03nm) was applied in probability density function and it could be used to describe the pore size distribution as well as effective mean pore radius of membranes

2.3. Electrokinetic Analyzer

Tangential streaming potential (TSP) were made using the SurPASS Electrokinetic Analyzer (Anton-Paar KG, Graz, Austria). The system having the pair of membrane area of 10 × 20 mm^2 was placed in the measuring cell. The measuring cell was separated by adjustable clamping cell (with the distance of 100±5µm) that formed a streaming channel. A background electrolyte of 1 mM KCl and 1 mM Na_2HCrO_4 solutions were used at room temperature with a target ramp pressure of 400 mbar; and the pH was adjusted in the range of pH 3–10 with 0.05 M HCl and 0.05 M KOH. The acid addition was carried out very slowly around the isoelectric point (*pI*) to ensure a good estimation of this point. Finally, once the isoelectric point reached in acidic range, the membrane samples were cleaned with deionized water and fresh electrolytes was used to calculate the zeta potential value in basic range. The TSP cell and the exact measuring procedure can be found elsewhere[34]. Many authors[41,42] had examined Helmholtz–Smoluchowski (H-S) correlation to determine the zeta potential value of the samples.

$$\zeta = \frac{\Delta U}{\Delta p} \times \frac{\eta}{\varepsilon \times \varepsilon_0} \times \kappa \qquad (6)$$

However, in this study, Fairbrother and Mastin (F-M) relationship (equation (6)) was used because it eliminated the surface conductance at low ionic strength[44].

3.0. Model Description.

The DSPM-DE model assumes that the membrane have porous, cylindrical structure and ions transport through the membrane occurred by diffusive mass transport, convective mass transport as well as electro-migration. A schematic of how convection, diffusion and electro-migration forces occur at the same time in negatively charged membrane is shown in Fig. 2.

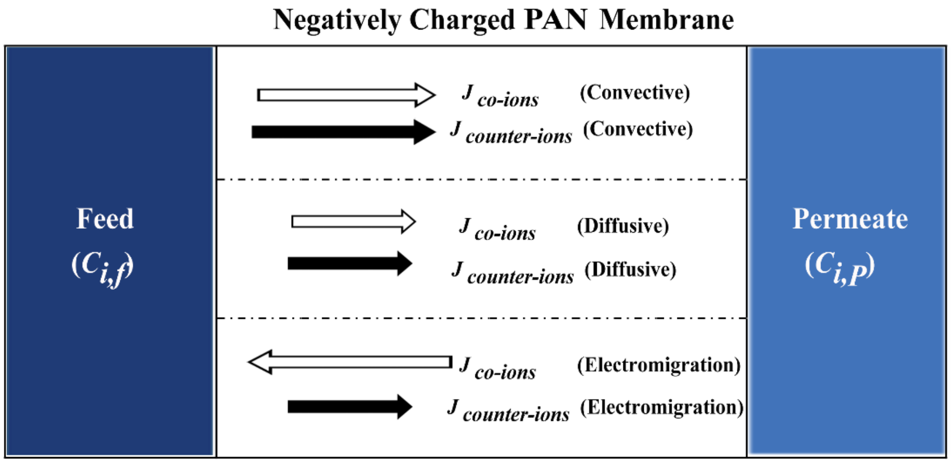

Figure 2: Schematic of convection, diffusion and electromigration forces on negatively charged membrane

As shown in Fig.2, the co-ions (anions) and counter ions (cations) showed the almost same effect on convective as well as diffusive mass transport from a pressure and concentration gradient. However, due to the electromigration the permeation of co-ions is lesser than counter ions. It is due to Donnan potential effect in which counter ions are attracted in negative charge membrane whereas co-ions are repulsed. Thus the permeation of co-ions were less as compared to counter ions. It is also noted that all driving forces were in the direction of permeate side with the exception of the electromigration of co-ions oriented towards the feed solution. Moreover, any electrolyte, the sum of the total co-ions flux must be equal to the total counter-ion flux so as to maintain the electroneutrality conditions. The following equation (7) based on extended Nernst-Planck (ENP) for the process prediction of solute flux was used.

$$J_i = K_{i,c} c_i V + \left(-D_{i,p} \frac{dc_i}{dx}\right) + \left(-z_i c_i D_{i,p} \frac{F}{RT} \frac{d\psi}{\mathrm{dx}}\right) \quad (7)$$

Where, $D_{i,p} = K_{i,d} D_{i,\infty}(\eta_o/\eta)$ i-th ion diffusion coefficient within pores and it can be affected by the change in viscosity within the pore.

3.1. Model validation.

The model aim was to obtain solute concentrations in permeate ($C_{i,p}$) from known solute concentrations at the feed wall ($C'_{i,f}$) as a function of flux. The DSPM-DE model described the interaction between the membrane and ionic solutes. Initially, the steric hindrance and Donnan equilibrium were used to determine the equilibrium partitioning between membrane and external (feed) solutions. Later, it incorporated dielectric exclusion (DE) principle as an additional electrostatic partitioning effect at the interfaces between the pore and external solution. A schematic diagram of transport mechanisms of chromate ions via hydrolyzed PAN membrane shown in Fig. 3. and the nomenclature of model parameters can be found elsewhere[34].

$$\frac{dc_{i,f}\,(x)}{dx} = \frac{V}{D_{i,p}}\left(K_{i,c} c_{i,f}(x) - C_{i,p}\right) - \frac{z_i c_{i,f}(x)}{RT} F \frac{d\psi}{\mathrm{dx}} \quad (8)$$

In addition, the ions transport through the membrane (equation (8)) derived by Runge-Kutta-Gill method using Matlab program and numerical scheme for computing rejection of i-th ion (R_i) described elsewhere [27].

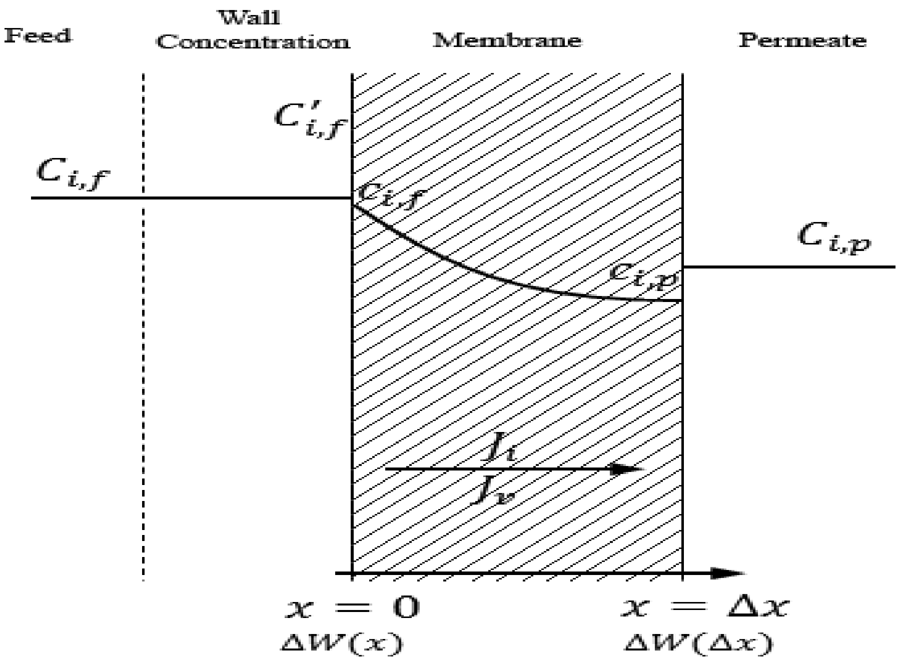

Figure 3: Transport mechanism of DSPM-DE model for Ions transport via negatively charged PAN UF membrane

Generally, dielectric exclusion occurs due to the difference between the dielectric properties of solute within the pore and the aqueous (bulk) solution. Due to the nano-sized pores, the dielectric constant within the pore is lesser than the bulk aqueous solution[35]. It was reported the dielectric constant of the pore (ε_p) can be calculated by two different methods. The first, true rejection coefficient data were used to determine the ε_p with fitting procedure. Later, the volumetric charge density (X_d) was applied to evaluate ε_p by adsorption isotherm[44]. In this study, the ε_p was calculated by using adsorption isotherm. The experimental studies also proved that X_d depends on the ion concentration of the solution i.e., when salt concentration (Na_2CrO_4) increased, the charge density also increased monotonously. Thus, it can be described by the adsorption isotherm (equation (9))[45].

$$|X_d| = q\left(C_f'\right)^s \sqrt{\frac{\varepsilon_p}{\varepsilon_b}} \qquad (9)$$

The adsorption parameters q = 1.57 and s = 0.57 estimated by Freundlich isotherm and these values were calculated by logarithmic form of X_d versus C_f'. The obtained values were corrected by $\sqrt{\varepsilon_p/\varepsilon_b}$ to fulfill the dielectric constant of inside the pore (ε_p) . For this study, the ε_p was kept constant at value of 48.98 and the remaining parameters were changed to study the chromate ion flux as followed.

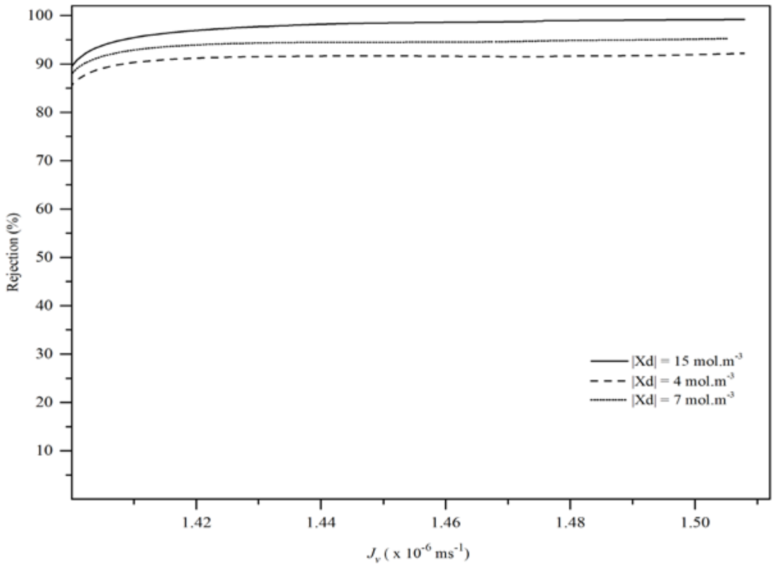

Figure 4: Variation of the rejection coefficient of chromate as a function of J_v (for different volumetric charge density (X_d)) at 0.67 mol.m^{-3} of Cr (VI), temperature: 27^{0}C, pressure 200 kPa (Simulated data from DSPM-DE).

As shown in Fig. 4, the single model parameter $\Delta x/A_k$ (1.4 x 10^{-5} m) and effective mean pore radius r_p (2.01 x 10^{-9} m) was used to obtain the rejection properties of chromate ion with respect to charge density of the membrane. The highest rejection coefficient (90%) was obtained in the range of $|X_d|$ value of 4 mol.m^{-3} to 15 mol.m^{-3}; which implied membrane had highly negative charge and it repelled the anionic solutes. This behavior could be explained by the electrostatic interaction between the solute and surface charge of the membrane. Some calculations were also done for the percentage of rejection coefficient with the function of membrane thickness to the porosity ($\Delta x/A_k$).

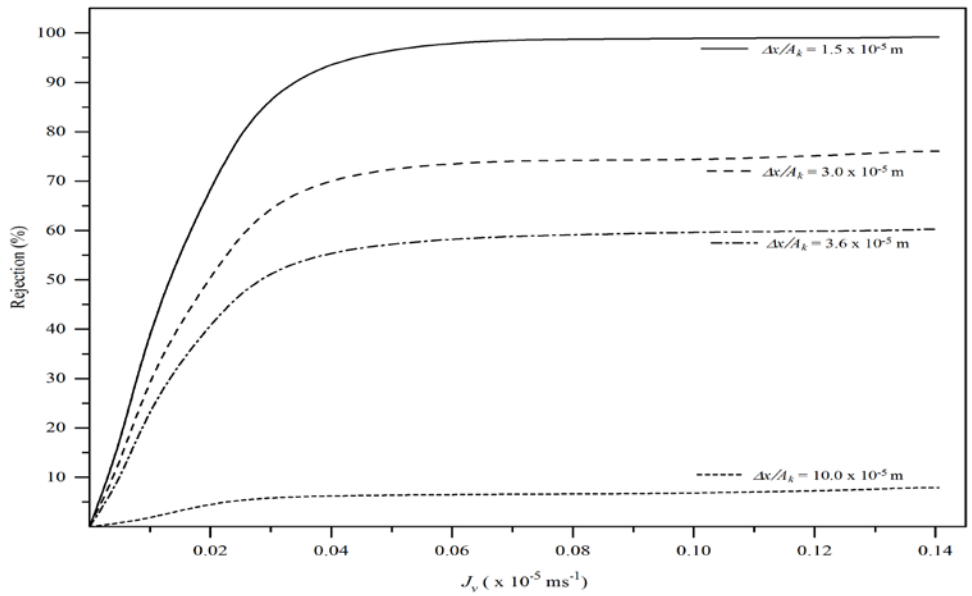

Figure 5: Effect of rejection coefficient of Cr (VI) (for different value of $\Delta x/A_k$) as a function of J_v for 0.67 mol.m^{-3} of feed concentration, temperature: 27^0C, pressure 200 kPa. (Simulated data from DSPM-DE).

As shown in Fig. 5, at high value of $\Delta x/A_k$ (10.0 x 10^{-5}m) the rejection coefficient was 10 %. Nevertheless, the rejection coefficient increased (≥ 94 %) with the decrease of membrane thickness to the porosity value (1.5 x 10^{-5}m). It confirmed that effective thickness to porosity of the membrane played an important role in the transport of ions through the membrane. It can be concluded, the rejection properties were directly proportional to the volumetric charge density and inversely to the ratio between thickness and porosity ($\Delta x/A_k$) of the membrane.
